# Seeing clearly with CLARI-O: a window into cellular architecture, interactions, and morphology of organoid models

**DOI:** 10.3389/fnins.2026.1874430

**Published:** 2026-07-28

**Authors:** Samra Beyene, Martin Thunemann, Natalie Baker Campbell, Elizabeth K. Kharitonova, Shira Klorfeld-Auslender, Farzad Mortazavi, Ella Zeldich

**Affiliations:** 1 Department of Anatomy and Neurobiology, Boston University Chobanian and Avedisian School of Medicine, Boston University, Boston, MA, United States; 2 Department of Medicine, Boston University Chobanian and Avedisian School of Medicine, Boston University, Boston, MA, United States; 3 Department of Biomedical Engineering, Boston University, Boston, MA, United States; 4 Neurophotonics Center, Boston University, Boston, MA, United States; 5 Picower Institute for Learning and Memory, Massachusetts Institute of Technology, Cambridge, MA, United States; 6 Center for Systems Neuroscience, Boston University, Boston, MA, United States

**Keywords:** assembloids, CLARITY, neuronal activity, organoid vascularization, organoids, tissue clearing, transplanted organoids, xenotranplantations

## Abstract

Cortical organoids (COs) represent a powerful *in vitro* model system that recapitulates key aspects of human brain development, enabling the study of neurodevelopmental processes, cellular diversity, and disease mechanisms in a physiologically relevant 3D environment. However, traditional histological analysis of COs relies on tissue sectioning, which limits the ability to capture the full spatial complexity of organoid architecture. In this study, we establish a framework for applying CLARI-O, an improved tissue-clearing technique, for intact COs and organoid-based systems, enabling comprehensive 3D visualization and analysis of 3D organizational features. Using CLARI-O in combination with high-resolution imaging, we demonstrate the utility of tissue clearing for studying glial populations, including oligodendrocytes and microglia, considered to be underrepresented in COs, and their interactions with neurons. Additionally, we apply this method to forebrain assembloids (FAs) to visualize cellular heterogeneity and the interface between ventral and dorsal regions. Finally, we use CLARI-O to study mouse brains containing xenotransplanted COs (MB-COs) to evaluate human cell integration, migration, vascularization, and structural connectivity. This is the first study to demonstrate how tissue clearing can be used after functional assays such as calcium imaging to correlate neural activity with *post hoc* structural analysis in MB-COs. Together, this work establishes CLARI-O as a powerful tool for advancing 3D structural and functional interrogation of human CO-derived systems, enhancing their value for disease modeling, drug screening, and translational neuroscience.

## Graphical abstract

**Figure:**
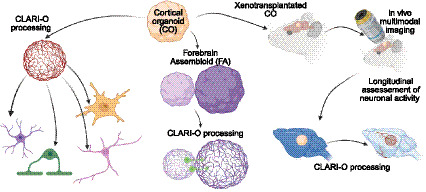
CLARI-O enables integrated structural analysis of organoids, assembloids, and xenografts. Graphical abstract was created with BioRender.com (agreement number DL29WNOR1V).

## Highlights

- We optimized clearing protocols to develop an organoid specific clearing method (CLARI-O) that enables high-resolution visualization of diverse neuronal and glial populations without tissue sectioning, preserving long-range connections and cellular processes.
- Forebrain assembloids used to study neuronal and oligodendrocyte migration can be effectively processed using CLARI-O, allowing detailed visualization of fusion interface.
- We established a robust framework for CLARI-O-based clearing of mouse brain tissue containing xenotransplanted human cortical organoids, enabling comprehensive 3D analysis of graft development, integration, and vascularization *in vivo*.

## 1 Introduction

The three-dimensional (3D) nature of human cortical organoids (COs) uniquely offers a holistic view of developmental pathways, cellular kinetics and interactions, connectivity, and functional dynamics in a single model. COs are generated through self-assembly of either embryonic stem cells or induced pluripotent stem cells (iPSCs), including patient-derived iPSCs. These COs can be regionally patterned to represent more region-specific cell types, which in turn form organized structures composed of progenitor, neuronal, and glial cell types. Such cells transcriptionally and electrophysiologically resemble mid-term gestation human prenatal brains (Lancaster et al., 2017; Lancaster et al., 2013; Qian et al., 2017; Kadoshima et al., 2013; Pasca et al., 2015; Bershteyn et al., 2017; Kang et al., 2024; Fair et al., 2020). Within these 3D platforms, multiple cell lineages differentiate, migrate, mature, and interact with each other. This allows for researchers to study the intricate dynamics of tissue organization, development, and function in ways that are not possible in traditional 2D cultures. Recent advances have further extended the utility of COs by enabling their xenotransplantation into rodent brains, where human cells achieve unprecedented levels of maturation and functional integration into host cortical circuits (Wilson et al., 2022; Revah et al., 2022).

A widely used method for characterizing organoid morphology and cell composition is immunohistochemical analysis of fixed serial sections (Pasca et al., 2015; Sloan et al., 2018; Madhavan et al., 2018; Li et al., 2022). This traditional histological approach relies on thin slicing of tissue, which offers only a limited, two-dimensional view, thus, offering a limited representation of the organoid-wide neural networks, structural connectivity, and complex internal organization. In addition, mechanical sectioning inevitably severs axonal projections and long-range processes, obscuring the true three-dimensional architecture of neural circuits. Furthermore, the effectiveness of immunohistochemical analysis can be compromised by the low permeability of antibodies and potential tissue damage during sectioning. These limitations restrict our ability to take full advantage of the complex cytoarchitecture and spatial arrangements within COs *in vitro* and mouse brains containing xenotransplanted COs (MB-COs) *ex-vivo*. To overcome these limitations, a sectioning-free, whole-organoid imaging platform is needed.

Tissue clearing techniques have significantly advanced organoid research by allowing scientists to visualize the intricate 3D structures within these miniaturized organ models (Shnaider and Pristyazhnyuk, 2021; Susaki and Takasato, 2021). By removing lipids that cause light scattering, tissue clearing makes entire organoids transparent, while preserving proteins and overall structural integrity (Vieites-Prado and Renier, 2021), thus enabling complete, three-dimensional imaging and reconstruction without the need for physical sectioning. This allows for the visualization of immunofluorescently labeled membrane, synaptic, signaling, and lineage-specific marker proteins. We had previously published a detailed clearing protocol in 2019 (Mortazavi et al., 2019), which we validated in zebrafish, mouse, rat, non-human primate, and human brain samples. Here, we describe how we have optimized this protocol and developed CLARI-O, an organoid-specific clearing protocol that maintains structural integrity, preserves cell-specific fluorescent labeling, and enables repeated imaging of the same sample. Specifically, we adapted the 2019 protocol (Mortazavi et al., 2019) by incorporating low-speed centrifugation and extended heat-assisted passive clearing into the workflow, which improved clearing uniformity across samples of varying size and complexity while minimizing mechanical disruption. Furthermore, these refinements improved reagent penetration while preserving tissue integrity and endogenous fluorescence, ensuring high-fidelity imaging across multiple rounds of imaging and analysis.

CLARI-O was used to clear COs, forebrain assembloids (FAs), and MB-COs, enabling high-resolution, three-dimensional analysis of organoid architecture and the longitudinal integration of CO-derived human cells within the mouse brain following *in-vivo* functional assays. Using CLARI-O, we were able to visualize not only the canonical cell populations present in dorsally derived organoids, such as neurons and astrocytes, but also cell types not typically represented in classical CO models. Here, we demonstrated that our CLARI-O approach allows for the visualization of distinct niches of oligodendrocytes that emerge following expansion of glial progenitors, and exogenously incorporated iPSC-derived microglia, as well as the detailed interactions between neuron and glia cells. Because these interactions are spatially organized, preserving intact three-dimensional structure is essential for accurate interpretation.

Furthermore, xenotransplantation of COs into the mouse cortex provides a maturation-promoting environment that supports vascularization and enables a more comprehensive assessment of neuronal activity. Following implantation of virally labeled cells and *in vivo* calcium imaging, the host mouse brains were subsequently cleared to allow volumetric analysis of the graft and surrounding tissue. CLARI-O–based imaging enabled visualization of the overall MB-CO architecture, its vascular integration, and the spatial distribution of human cells within the host brain. Together, the integration of functional live-cell imaging with high-resolution 3D structural analysis through CLARI-O provides a powerful and complementary strategy to dissect structure–function relationships, elucidate disease mechanisms, evaluate therapeutic interventions, and advance next-generation models of human brain development.

Altogether, this study establishes a robust and scalable framework for leveraging CLARI-O to comprehensively visualize and interrogate the structural organization, cellular diversity, and integrative complexity of COs and MB-COs within their intact three-dimensional environment.

## 2 STAR methods

### 2.1 Materials and equipment

#### 2.1.1 Cell lines

- Human iPSCs (WC-24-02-DS-B; female, normal karyotype)
- Human iPSCs (MGH2046) (Seo et al., 2017) kindly provided by Dr. Steven J. Haggarty (Harvard Medical School and Massachusetts General Hospital).

#### 2.1.2 Cell culture reagents

- Matrigel (Corning, cat. 354,277)
- mTeSR Plus (STEMCELL Technologies, cat. 85850)
- ReLeSR (STEMCELL Technologies, cat. 85850)
- Accutase (STEMCELL Technologies, cat. 7920)
- ROCK inhibitor (Fisher Scientific, cat. 12-541-0)

#### 2.1.3 Basal media and supplements

- DMEM/F12 (ThermoFisher Scientific, cat. 11320-033)
- Neurobasal-A medium (ThermoFisher Scientific, cat. 10888022)
- B-27 supplement minus vitamin A (ThermoFisher Scientific, cat. 12587)
- N2 supplement (ThermoFisher Scientific, cat. 17502048)
- GlutaMAX (ThermoFisher Scientific, cat. 35050061)
- Penicillin/Streptomycin (ThermoFisher Scientific, cat. 15140122)
- Primocin (Invivogen, cat. Ant-pm-1)
- MEM NEAA (ThermoFisher Scientific, cat. 11140-050)

#### 2.1.4 Patterning molecules and growth factors

- Dorsomorphin (MilliporeSigma, cat. P5499)
- SB-431542 (MilliporeSigma, cat. S4317)
- FGF2 (R&D Systems, cat. 233-FB-25/CF)
- EGF (R&D Systems, cat. 236-EG-200)
- PDGF-AA (R&D Systems, cat. 221-AA)
- IGF (R&D Systems, cat. 291-GF-200)
- T3 (MilliporeSigma, cat. T6397)
- BDNF (PeproTech, cat. AF-450-02)
- NT3 (PeproTech, cat. 450-03)
- IWP-2 (Selleckchem, cat. S7085)
- SAG (MilliporeSigma, cat. 566660)
- HGF (PeproTech, cat. 315-23)
- cAMP (MilliporeSigma, cat. D0627)
- Biotin (MilliporeSigma, cat. B4639)
- Ascorbic acid (MilliporeSigma, cat. A4403)

#### 2.1.5 Tissue culture plastics

- Low-adhesion V-bottom 96-well plates (S-Bio Prime, cat. MS-9096VZ)
- Ultra-low attachment 24-well plates (ThermoFisher Scientific, cat. 07-200-602)

#### 2.1.6 CLARI-O clearing reagents

- Sodium dodecyl sulfate (SDS) (Bio-Rad, cat. 1610302)
- Lithium hydroxide monohydrate (Fisher Scientific, cat. L127500)
- Boric acid (MilliporeSigma, cat. B2645)
- Sodium hydroxide (MilliporeSigma, cat. 901915)
- Tris-buffered saline (TBS) (MilliporeSigma, cat. PPB011)

#### 2.1.7 Immunostaining reagents

- Triton X-100 (MilliporeSigma, cat. X100)

#### 2.1.8 Primary antibodies

- Anti-GABA (Sigma, cat. A2052, RRID: AB_477652)
- Anti-GFAP (Abcam, cat. ab4674, RRID: AB_304558)
- Anti-GFAP-AF594 (Novus Biologicals, cat. NBP1-05197AF594, RRID: AB_3198395)
- Anti-Iba1 (Aves Labs, cat. IBA1, RRID: AB_2910556)
- Anti-MAP2 (Synaptic Systems, cat. 188004, RRID: AB_2138181)
- Anti-NeuN-AF594 (Novus Biologicals, cat. NBP1-77686AF594)
- Anti-NFH-AF488 (Novus Biologicals, cat. NB500-416AF488, RRID: AB_3191074)
- Anti-MBP-AF488 (Novus Biologicals, cat. NBP2-22121AF488, RRID: AB_2885126)
- Anti-PSD95 (ThermoFisher Scientific, cat. 51-6,900, 2533914)
- Anti-Synaptophysin (MilliporeSigma, cat. MAB368, RRID: AB_94947)

#### 2.1.9 Secondary antibodies

- Donkey anti-mouse AF488 (ThermoFisher, cat. A-21202, RRID: AB_141607)
- Goat anti-rabbit AF568 (ThermoFisher, cat. A-11011, RRID: AB_143157)
- Donkey anti-rabbit AF594 (ThermoFisher, cat. A-21207, RRID: AB_141637)
- Goat anti-guinea pig AF647 (ThermoFisher, cat. A-21450, RRID: AB_2535867)
- Donkey anti-goat AF647 (Abcam, cat. ab150135, RRID: AB_2687955)

#### 2.1.10 Additional reagents

- Glycerol (ThermoFisher, cat. EP229)
- DyLight 649 Tomato Lectin (Vector Laboratories, cat. DL11781)

#### 2.1.11 Equipment

- Temperature-controlled water bath (37 °C)
- Centrifuge (capable of 1,500–4,000 rpm, temperature control)
- Orbital shaker
- Confocal microscope (e.g., Leica SP8)
- Two-photon microscope (Bruker Ultima or equivalent)
- Imaging software (LAS X, Imaris)

### 2.2 Methods

#### 2.2.1 Maintenance and preparation of human iPSCs


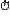
 Timing: ongoing:

Human induced pluripotent stem cells (iPSCs) are maintained under feeder-free conditions prior to organoid generation.

1. Use a female iPSC line with a normal karyotype (WC-24-02-DS-B), previously characterized.
2. Culture cells on Matrigel-coated plates (Cat# 354277, Corning).
3. Maintain cells in mTeSR Plus medium (Cat# 85850, STEMCELL Technologies).
4. Change media every other day.
5. Passage cells using ReLeSR (Cat# 85850, STEMCELL Technologies) according to manufacturer instructions.
6. Maintain cells within passages 24–50.
7. Ensure normal morphology prior to use for organoid generation.

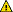
 Critical: Only use iPSC cultures with uniform colony morphology and no signs of spontaneous differentiation.


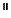
 Pause point: iPSCs can be maintained continuously under these conditions until ready for differentiation.

#### 2.2.2 Generation of dorsal cortical organoids (dCOs)

The generation of dCOs is based on Pasca et al. (2015).

##### 2.2.2.1 Preparation and dissociation of iPSCs


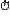
 Timing: ~30–40 min:

1) Aspirate media from one well of a 6-well plate of iPSCs (prepared as in Section 1).
2) Wash cells with 1 mL PBS.
3) Add 1 mL Accutase (Cat# 7920).
4) Incubate for ~5 min at 37 °C.
5) Gently triturate to obtain a single-cell suspension.
6) Add 5 mL DMEM/F12 (Cat# 11330-057).
7) Centrifuge for 5 min.
8) Resuspend pellet in:

- 1 mL mTeSR Plus
- 50 μM ROCK inhibitor (Cat# 12–541-0)

9) Count cells.

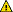
 Critical: Avoid excessive trituration to maintain cell viability.

##### 2.2.2.2 Seeding and embryoid body formation (day 0)


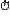
 Timing: ~1 h:

1) Plate 15,000 cells per well in 150 μL media.
2) Use low-adhesion V-bottom 96-well plates (Cat# MS-9096VZ).
3) Incubate at 37 °C, 5% CO₂.

##### 2.2.2.3 Neural induction (days 1–6)


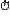
 Timing: 6 days:

Day 1:

1) Replace media with 100 μL TeSR-E6 (Cat# 0596) supplemented with:
  - 2.5 μM Dorsomorphin (Cat# P5499)
  - 10 μM SB-431542 (Cat# S4317)

Days 3–6:

1) Perform daily full media replacement with fresh supplemented TeSR-E6.

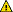
 Critical: Perform gentle media changes to avoid disrupting aggregates.

##### 2.2.2.4 Transition to neural media (day 7)


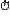
 Timing: ~30 min:

1) Replace media with 100 μL Neural Media (NM) containing:
  - Neurobasal-A (Cat# 10888022)
  - B-27 minus vitamin A (1:50; Cat# 12587)
  - GlutaMAX (1:100; Cat# 35050061)
  - Penicillin/Streptomycin (1:100; Cat# 15140122)
  - Primocin (1:500; Cat# ant-pm-1)
2) Supplement with:
  - FGF2 (20 ng/mL; Cat# 233-FB-25/CF)
  - EGF (20 ng/mL; Cat# 236-EG-200)

##### 2.2.2.5 Neural progenitor expansion (days 7–25)


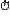
 Timing: ~2.5 weeks:

1) Maintain organoids in NM + FGF2 + EGF.
2) Perform media changes every 2–3 days.

##### 2.2.2.6 Transfer to 24-well plates (days 16–24)


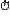
 Timing: ~30–60 min:

1) Transfer organoids individually to ultra-low attachment 24-well plates (Cat# 07–200-602).

##### 2.2.2.7 Neuronal maturation phase (days 25–39)


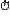
 Timing: ~2 weeks:

1) Culture in NM supplemented with:
  - BDNF (20 ng/mL; Cat# AF-450-02)
  - NT3 (20 ng/mL; Cat# 450–03)
  - Geltrex (1%; Cat# A1569601)
2) Perform half-media changes every other day.

##### 2.2.2.8 Long-term maintenance (day 40 onward)


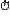
 Timing: ongoing:

1) Maintain in NM + 1% Geltrex.
2) Perform half-media changes every 2 days.

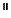
 Pause point: Organoids can be maintained long-term (>100 days).

#### 2.2.3 Generation of oligodendrocyte-enriched organoids (dOCOs)

The generation of dOCOs is based on Madhavan et al. (2018).

##### 2.2.3.1 Initial organoid generation


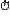
 Timing: Days 0–50:

dOCOs are generated following the procedure described in Section 2.1–2.8, with identical conditions maintained until Day 50.

##### 2.2.3.2 Oligodendrocyte expansion (days 50–60)


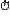
 Timing: 10 days:

1) Supplement NM with:
  - PDGF-AA (10 ng/mL; Cat# 221-AA)
  - IGF (10 ng/mL; Cat# 291-GF-200)

##### 2.2.3.3 Oligodendrocyte differentiation (days 61–69)


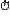
 Timing: ~9 days:

1) Add:
  - T3 (40 ng/mL; Cat# T6397)
2) Perform media changes every 2 days.

##### 2.2.3.4 Maintenance (day 70 onward)


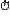
 Timing: ongoing:

1) Maintain organoids in NM.
2) Change media every 2 days.

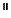
 Pause point: dOCOs can be used for downstream applications, including assembloid formation (Section 5).

#### 2.2.4 Generation of ventral cortical organoids (vCOs)

The generation of vCOs is based on Marton et al. (2019).

##### 2.2.4.1 Initial steps


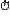
 Timing: Days 0–6:

vCOs are generated following Section 2.1–2.3, with the addition of:

- IWP-2 (5 μM; Cat# S7085) between Days 4–24

##### 2.2.4.2 Ventral patterning (days 12–24)

1) Add:
  - SAG (1 μM; Cat# 566660)

##### 2.2.4.3 Transition to differentiation medium (day 7 onward)

1) Replace with differentiation and maintenance medium (DMM) containing:
  - DMEM/F12 (Cat#11320–033)
  - B-27 minus vitamin A (1:50; Cat# 12587)
  - N2 (1:100, Cat# 17502048)
  - Insulin (25 μg/mL; Cat# I9278-5ML)
  - MEM NEAA (1:100; Cat#11140–050)
  - Penicillin/Streptomycin (1:100; Cat# 15140122)
  - GlutaMAX (1:100; Cat# 35050061)
  - *β*-mercaptoethanol (0.1 mM; cat. Cat# M3148)
2) Supplement with:
  - FGF2 + EGF (Days 7–24)

##### 2.2.4.4 Neuronal and oligodendrocyte enrichment (days 24–37)

1) Supplement DMM with:
  - BDNF (20 ng/mL; Cat# AF-450-02)
  - NT3 (20 ng/mL; Cat# 450–03)
  - PDGF-AA (10 ng/mL; Cat# 221-AA)
  - IGF (10 ng/mL; Cat# 291-GF-200)
  - T3 (60 ng/mL; Cat# T6397)
  - HGF (5 ng/mL; Cut315-23)
  - cAMP (1 μM; cat. D0627)
  - Biotin (100 ng/mL; cat. B4639)

##### 2.2.4.5 Long-term maturation (day 37 onward)

1) Maintain in DMM supplemented with:
  - T3 (60 ng/mL; Cat# T6397)
  - cAMP (1 μM; cat. D0627)
  - Biotin (100 ng/mL; Cat# B4639)
  - Ascorbic acid (20 μg/mL; Cat# A4403)
2) Change media twice weekly.

#### 2.2.5 Generation of forebrain assembloids (FAs)

##### 2.2.5.1 Fusion (day 70)


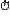
 Timing: 3 days:

1) Combine vCOs (Section 4) and dCOs/dOCOs (Sections 2–3) in microcentrifuge tubes.
2) Add 1 mL NM media.
3) Incubate for 3 days.

##### 2.2.5.2 Maintenance of FAs


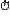
 Timing: ongoing:

1) Perform half-media change on Day 2 of fusion.
2) Transfer to 24-well plates.
3) Continue half-media changes every other day.

#### 2.2.6 Lenti virus (LV) labeling of dorsal cortical organoids (dCOs)

The Lenti virus (LV) labeling of dCOs is based on Revah et al. (2022).

##### 2.2.6.1 Preparation of organoids for labeling


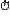
 Timing: ~30 min:

1) Select dCOs generated as described in Section 2 at Day 40 *in vitro* (DIV).
2) Transfer individual organoids into 1.5 mL microcentrifuge tubes.

##### 2.2.6.2 Lentiviral transduction


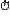
 Timing: overnight incubation:

1) Prepare viral mixture containing:
  - 2 μL pLV-Bls-EF1A > mScarlet3 (Cat# VB240919-1466xpw)
  - 4 μL pLV-Puro-SYN1.jGCaMP8s (Cat# VB240904-1449tnm)
  - ~20 μL maintenance media
2) Add viral mixture to each organoid.
3) Incubate at 37 °C for 30 min.
4) Add 300 μL maintenance media.
5) Incubate overnight at 37 °C.

##### 2.2.6.3 Post-labeling maintenance


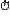
 Timing: ongoing:

1) Transfer organoids back to 24-well plates.
2) Maintain under standard conditions (Section 2.8).
3) Verify fluorescence expression prior to downstream use.

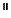
 Pause point: Labeled organoids can be maintained until xenotransplantation (Section 9).

#### 2.2.7 Generation of human microglia

These steps of the protocol are based on the protocol associated with STEMdiff Hematopoietic Kit (Cat# 05310) and STEMdiff Microglia Differentiation Kit (Cat# 100-0019).

##### 2.2.7.1 Differentiation to hematopoietic progenitor cells (HPCs)


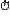
 Timing: 12 days:

1) Differentiate iPSCs (Section 1) using:
  - STEMdiff Hematopoietic Kit (Cat# 05310)
2) On Day 12:
  - Collect HPCs
  - Validate expression of CD43, CD45, and CD34 by flow cytometry

##### 2.2.7.2 Cryopreservation of HPCs


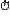
 Timing: ~1 h:

1) Resuspend 700,000 HPCs in:
  - 1 mL freezing media (Cat# 05859)
2) Cryopreserve according to standard procedures

##### 2.2.7.3 Differentiation into microglia


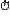
 Timing: 24 days:

1) Thaw 200,000 HPCs.
2) Differentiate using:
  - STEMdiff Microglia Differentiation Kit (Cat# 100–0019)
3) Culture for 24 days.
4) Validate microglial identity:
  - CD45, CD11b, TREM2 expression
  - ≥80% CD45+/CD11b + cells

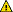
 Critical: Low differentiation efficiency may impact downstream incorporation (Section 8).

#### 2.2.8 Incorporation of microglia into COs

The incorporation of the microglia into COs is based on Schafer et al. (2023). Microglia was incorporated into the organoids on day 110.

##### 2.2.8.1 Co-culture setup


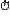
 Timing: 3 days:

1) Select Day 50 COs (Section 2 or 3).
2) Transfer a single organoid into an Eppendorf tube with carryover media.
3) Add:
  - 50,000 microglia
  - 100 μL microglia differentiation media
4) Incubate for 3 days at 37 °C to allow microglial attachment and migration.

##### 2.2.8.2 Post-incorporation culture


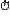
 Timing: 8 days:

1) Transfer organoids to ultra-low attachment 24-well plates.
2) Culture in Neural Media (Section 2.4) + 1% Geltrex.
3) Supplement with:
  - STEMdiff Microglia Supplement 2 (1:250; Cat# 100–0023)
4) Maintain for 8 days.

##### 2.2.8.3 Fixation


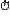
 Timing: 2 days:

1) Fix organoids in:
  - 4% PFA (Cat# P6148) for 48 h

#### 2.2.9 Xenotransplantation and *in vivo* imaging


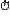
 Timing: multi-day procedure (surgery + recovery + imaging):

Due to the technical complexity and length of the xenotransplantation and *in vivo* imaging procedures, a detailed step-by-step description is beyond the scope of this protocol. For comprehensive methodology, including surgical procedures, perioperative care, and two-photon imaging acquisition and analysis, please refer to our previous publication (Wilson et al., 2022). Experiments and animal procedures were conducted according to the Guide for the Care and Use of Laboratory Animals and protocols approved by the Boston University Institutional Animal Care and Use Committee (PROTO202000026). We used non-obese diabetic, severe combined immunodeficient (NOD/SCID) mice (Charles River Laboratories) at an age of 12–20 weeks that were single- or group-housed in isolated ventilated rodent cages under normal light–dark cycles with nestlet enrichment and unrestricted access to food and water.

##### 2.2.9.1 Overview of xenotransplantation

1) dCOs generated as described in Sections 2–6 are transplanted into the retrosplenial cortex of immunodeficient mice.
2) The procedure includes:
  - Craniotomy and durectomy
  - Local cortical aspiration (~1 mm^3^ cavity)
  - Placement of a single organoid
  - Sealing with a cranial window

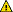
 Critical: Ensure consistency in implantation depth and cavity size to enable reproducible integration and imaging.

##### 2.2.9.2 Post-operative care and recovery


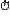
 Timing: ~7 days:

1) Administer analgesics and antibiotics according to institutional guidelines.
2) Allow animals to recover for at least 7 days prior to imaging.

##### 2.2.9.3 *In vivo* imaging overview

1) Animals are habituated to head fixation prior to imaging.
2) Two-photon imaging is performed in awake, head-fixed animals.
3) Calcium imaging and vascular imaging can be performed depending on experimental design.

##### 2.2.9.4 Data processing overview

1) Motion correction is performed using normcorre.
2) ROI extraction and signal analysis are performed using Suite2P.

#### 2.2.10 CLARI-O tissue clearing procedure

##### 2.2.10.1 Clearing of cortical organoids (COs) and forebrain assembloids (FAs)


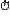
 Timing: ~15 days.

###### 2.2.10.1.1 Fixation of COs and FAs


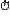
 Timing: 2 days:

1) Transfer COs or FAs into 15 mL Falcon tubes.
2) Wash once with PBS.
3) Add ~5 mL of 4% paraformaldehyde (PFA) to fully submerge samples.
4) Incubate at room temperature or 4 °C for 48 h.

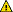
 Critical: Ensure complete immersion of samples to achieve uniform fixation (see Figure 1A).

**Figure 1:**
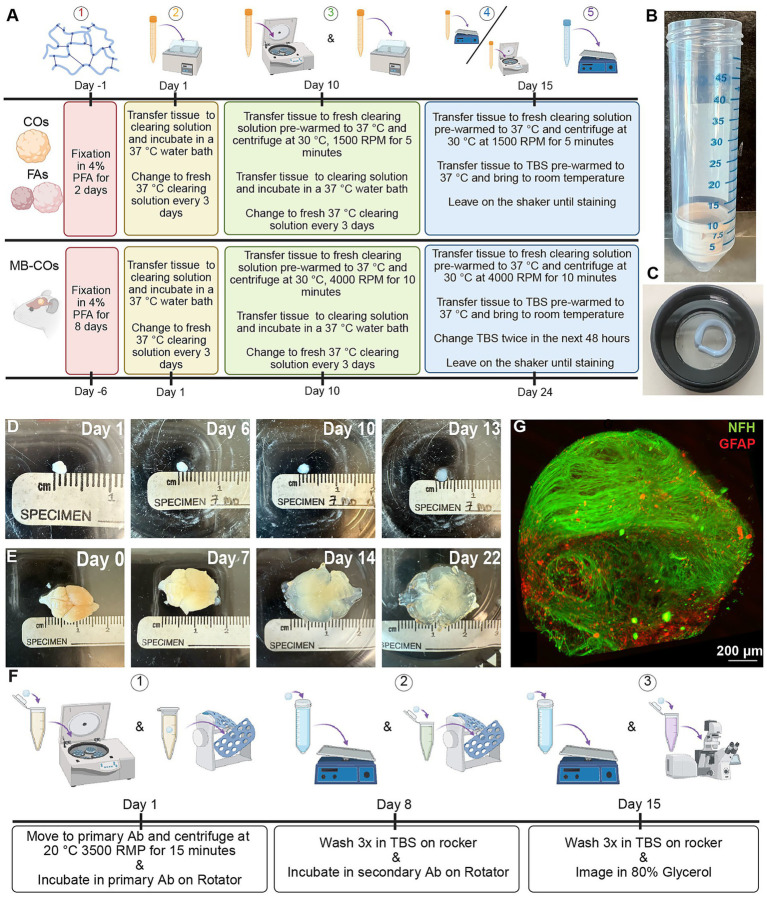
CLARI-O workflow for three-dimensional clearing and immunolabeling of human cortical organoids (COs), forebrain assembloids (FAs), and mouse brain-containing xenotransplanted cortical organoids (MB-COs). **(A)** Schematic overview of the CLARITY-O-clearing workflow optimized for COs, FAs, and MB-COs. COs, FAs, and MB-COs are first fixed in 4% paraformaldehyde, followed by incubation in clearing solution and sequential centrifugation steps to facilitate lipid removal and optical transparency. Samples are maintained at 37 °C with periodic replacement of clearing solution until sufficient tissue clearing is achieved prior to immunostaining. Illustration created with BioRender.com. **(B)** Representative image of Falcon tube fitted with a mesh insert. **(C)** Example of an assembly of the two glass-bottom PELCO wells, secured with BluTack to maintain stability and prevent dehydration throughout image acquisition. **(D)** Time-course images illustrating progressive optical clearing of CO during the CLARITY-O protocol. **(E)** Time-course images of MB-COs showing progressive tissue clearing over the course of the protocol. **(F)** Schematic of the downstream immunostaining workflow. Cleared samples are incubated with primary antibodies following centrifugation-assisted antibody penetration, washed in TBS, incubated with secondary antibodies, and subsequently prepared for imaging. Illustration created with BioRender.com. **(G)** Representative three-dimensional confocal reconstruction of a cleared day 120 CO immunolabeled for NFH (green; axonal marker) and GFAP (red; astrocytic marker), demonstrating preservation of neuronal and glial architecture following CLARITY-O processing. Scale bar, 200 μm.

###### 2.2.10.1.2 Passive clearing (days 1–10)


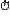
 Timing: 10 days:

CLARI-O solution:

- Boric acid (1 M, Cat# B2645) supplemented with:
- Sodium dodecyl sulfate (SDS; 200 mM, Cat# 1610302)
- Lithium hydroxide monohydrate (20 mM, Cat# L127500)
- Adjust the pH of the clearing solution to 9.0 using 0.1 M sodium hydroxide.

The solution is stable at room temperature for up to 6 months.

1) Transfer fixed samples into 15 mL Falcon tubes containing CLARI-O clearing solution (pre-warmed to 37 °C).
2) Place tubes in a 37 °C water bath.
3) Incubate for 10 days.
4) Replace clearing solution every 3 days with freshly prepared, pre-warmed (37 °C) solution.

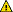
 Critical: Maintaining the clearing solution at 37 °C is essential—cold solution significantly slows lipid extraction.

###### 2.2.10.1.3 Centrifugation-assisted clearing (day 10)


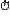
 Timing: ~30 min:

1) Transfer samples into fresh clearing solution (pre-warmed to 37 °C) in 15 mL Falcon tubes.
2) Centrifuge:
  - 230 × g
  - 5 min
  - 30 °C
3) Return samples immediately to 37 °C water bath in fresh clearing solution.

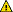
 Critical: Centrifugation improves penetration of clearing solution and accelerates optical clearing.

While swinging-bucket rotor centrifuge is not essential, our samples were centrifuged using an IEC Model CL-2 centrifuge equipped with a swinging-bucket rotor. Standard 15 mL conical tubes were maintained in the vertical orientation during centrifugation. Samples were centrifuged at 230 × g for 5 min at 30 °C using the instrument’s default acceleration and deceleration settings.

###### 2.2.10.1.4 Continued clearing (days 10–15)


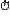
 Timing: 5 days

1) Continue incubation at 37 °C in clearing solution.
2) Replace solution every 3 days with freshly prepared, pre-warmed solution.

###### 2.2.10.1.5 Final centrifugation and transition to buffer (day 15)


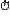
 Timing: ~30 min:

1) Transfer samples into fresh pre-warmed clearing solution.
2) Centrifuge:
  - 230 × g
  - 5 min
  - 30 °C
3) Immediately transfer samples into 0.05 M Tris-buffered saline (TBS, pre-warmed to 37 °C).
4) Allow samples to equilibrate to room temperature.

###### 2.2.10.1.6 Storage prior to immunostaining


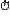
 Timing: up to 2 months:

1) Maintain samples in 0.05 M TBS at room temperature.
2) Place on a gentle shaker.

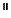
 Pause point: Cleared COs and FAs can be stored in TBS for up to 2 months prior to immunostaining.

##### 2.2.10.2 Clearing of mouse brains containing xenotransplanted cortical organoids (MB-COs)


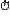
 Timing: ~24 days.

###### 2.2.10.2.1 Post-fixation of xenotransplanted brains


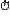
 Timing: 8 days:

1) Following transcardial perfusion, transfer whole brains into 50 mL Falcon tubes.
2) Add sufficient 4% paraformaldehyde (PFA) to fully submerge tissue (~30–40 mL).
3) Incubate at 4 °C for 8 days.

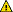
 Critical: Ensure complete immersion of tissue to achieve uniform fixation.

###### 2.2.10.2.2 Passive clearing (days 1–10)


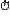
 Timing: 10 days

1) Transfer brains into 50 mL Falcon tubes containing ~30 mL of CLARI-O clearing solution (pre-warmed to 37 °C).
2) Place tubes in a 37 °C water bath.
3) Incubate for 10 days.
4) Replace clearing solution every 3–5 days with freshly prepared, pre-warmed (37 °C) solution.

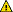
 Critical: Maintaining clearing solution at 37 °C is essential—cold solution significantly slows lipid extraction.

###### 2.2.10.2.3 Centrifugation-assisted clearing (day 10)


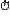
 Timing: ~1 h:

1) Transfer each brain into a 50 mL Falcon tube fitted with a mesh insert (Corning Netwell, Cat# CLS3519) (see Figure 1B).
2) Add 30 mL pre-warmed clearing solution.
3) Centrifuge:
  - 1700 × g
  - 10 min
  - 30 °C
4) Return samples immediately to the 37 °C water bath in fresh clearing solution.

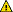
 Critical: The mesh insert suspends the tissue above the tube bottom, ensuring uniform detergent exposure during centrifugation.

###### 2.2.10.2.4 Continued clearing and monitoring (days 10–24)


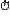
 Timing: ~14 days:

1) Continue incubation at 37 °C in clearing solution.
2) Replace solution every 3–5 days (pre-warmed).
3) Monitor tissue transparency daily.
4) Continue until desired optical transparency is achieved (typically Day 24).

###### 2.2.10.2.5 Final centrifugation and detergent removal


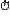
 Timing: ~30 min:

1) Transfer brains again into mesh insert-equipped 50 mL Falcon tubes.
2) Add 30 mL pre-warmed clearing solution.
3) Centrifuge:
  - 1700 × g
  - 10 min
4) This step removes residual detergent-rich clearing solution.

###### 2.2.10.2.6 Washing and equilibration


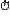
 Timing: 48 h:

1) Transfer brains into 50 mL Falcon tubes containing pre-warmed 0.05 M Tris-buffered saline (TBS).
2) Wash at room temperature for 48 h.
3) Replace TBS at least twice during this period (always pre-warmed before use).
4) Place samples on a gentle shaker during washing.

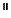
 Pause point: Cleared brains can be stored in 0.05 M TBS at room temperature on a shaker until immunostaining.

#### 2.2.11 Immunostaining and imaging of cleared samples

See Table 1.

**Table 1:** Primary and secondary antibodies used in each experiment.

Primary antibodies	Host species	Catalog number	Fluorophore	Incubat. volume	Sample type	Used in figure
anti-GABA (1:200)	Rabbit	Cat#A2052	AlexaFluor-594 (1:500)	1.5 ml	CO	Figures 3F,G
anti-GFAP (1:1000)	Chicken	Cat#ab4674	AlexaFluor-594 (1:1000)	1.5 ml	CO	Figure 1G
anti-GFAP (1:1000)	Mouse	Cat#NBP1-05197AF594	Pre-conjugated to AlexaFluor-594	1.5 ml	FA	Figures 3A–E
anti-Iba1 (1:500)	Chicken	Cat#IBA1	AlexaFluor-488 (1:1000)	1.5 ml	CO	Figures 2E–G
anti-MAP2 (1:500)	Guinea pig	Cat#188004	AlexaFluor-488 (1:1000)	1.5 ml	CO	Figure 2A,B
anti-NeuN (1:1000)	Rabbit	Cat#NBP1-77686AF594	Pre-conjugated to AlexaFluor-594 (1:1000)	1.5 ml	CO	Figures 2A,B
				1.5 ml	FA	Figures 3A,B
anti-NFH (1:500)	Mouse	Cat#NB500-416AF488	Pre-conjugated to AlexaFluor-488 (1:1000)	1.5 ml	CO	Figure 1G Figures 2C,D
anti-PSD95 (1:500)	Rabbit	Cat#51-6900	AlexaFluor-594 (1:1000)	1.5 ml	CO	Figure 2F,G
anti-Synaptophysin (1:500)	Mouse	Cat#MAB368	AlexaFluor-488 (1:1000)	1.5 ml	CO	Figure 3G
anti-MBP (1:500)	Mouse	Cat#NBP2-22121AF488	Pre-conjugated to AlexaFluor-488 (1:500)	1.5 ml	CO	Figures 2A,B
				1.5 ml	FA	Figures 3C,D


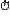
 Timing: ~14–15 days.

##### 2.2.11.1 Primary antibody incubation


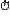
 Timing: 7 days:

1) Transfer cleared samples (Sections 10.1–10.2) into 1.5 mL microcentrifuge tubes.
2) Prepare primary antibody solution containing:
  - 0.05 M Tris-buffered saline (TBS), pH 7.6
  - 0.5% Triton X-100
3) Add ~1–1.5 mL of primary antibody solution to each tube.
4) Centrifuge samples:
  - 1,300 × g
  - 15 min
  - 20 °C
5) Incubate samples in primary antibody solution:
  - 7 days at room temperature
  - On a rotator (gentle agitation)

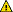
 Critical: Centrifugation prior to incubation significantly improves antibody penetration into cleared tissue (see Figure 1F, Day 1).

##### 2.2.11.2 Washing after primary antibody


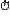
 Timing: ~30–60 min:

1) Transfer samples to 50 mL Falcon tubes containing 0.05 M TBS.
2) Wash 3 times for 10 min each.
3) Perform washes on a rocker at room temperature.

##### 2.2.11.3 Secondary antibody incubation


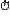
 Timing: 7 days:

1) Transfer samples back into 1.5 mL tubes.
2) Prepare secondary antibody solution:
  - 0.05 M TBS
  - 0.5% Triton X-100
3) Add ~1–1.5 mL of solution containing appropriate secondary antibodies.
4) Incubate:
  - 7 days at room temperature
  - On a rotator

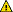
 Note: If using fluorophore-conjugated primary antibodies, this step can be omitted.

##### 2.2.11.4 Washing after secondary antibody


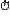
 Timing: ~30–60 min

1) Transfer samples to 50 mL Falcon tubes.
2) Wash 3 times for 10 min each in 0.05 M TBS.
3) Perform washes on a rocker at room temperature.

##### 2.2.11.5 Refractive index matching


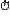
 Timing: ≥12–24 h

1) Transfer samples into:
  - 80% glycerol in 0.05 M TBS (pH 7.6)
2) Incubate until samples are fully equilibrated (typically overnight).

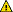
 Critical: Proper equilibration in glycerol is essential for refractive index matching. Residual refractive index heterogeneity within the specimen can lead to light scattering, optical aberrations, and signal attenuation. Immersion in 80% glycerol reduces these effects by matching the refractive index of the imaging medium to that of the cleared tissue, thereby increasing transparency, improving axial resolution, and enhancing visualization of cellular and subcellular structures.

##### 2.2.11.6 Imaging


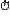
 Timing: 1–2 days:

1) Mount samples in 80% glycerol.
2) Position samples between glass-bottom wells and stabilize to prevent movement.
3) Perform imaging using a confocal microscope (e.g., Leica SP8) with:
  - Water immersion objective (e.g., 25×, high NA)
  - Deep z-stack acquisition
4) Optimize acquisition parameters (laser power, detector gain) per sample.
5) Perform:
  - Image stitching (if needed)
  - 3D reconstruction (e.g., LAS X, Imaris)

## 3 Results

### 3.1 CLARI-O enables rapid clearing and 3D imaging of intact COs and MB-COs

In 2019, Mortazavi et al. (2019) published a clearing method that not only allowed for the processing of whole rodent brains within 12 days but also proved being effective across multiple tissue types including human, non-human primate, and zebrafish brain. In this study, we further optimized Mortazavi’s approach by adapting it for use in COs, FAs, and MB-COs. A diagram of our organoid-optimized protocol, termed CLARI-O, is shown in Figure 1A, which provides a schematic timeline of this protocol, illustrating how the duration of each processing step is optimized according to whether it is a CO/FA, or MB-CO.

COs and FAs underwent fixation in 4% PFA for 2 days while MB-COs were fixed for 8 days. Following this, samples were incubated in 37 °C clearing solution for 10 days with fresh pre-warmed solution changes every 3 days. We prioritized passive clearing over electrophoretic tissue clearing (ETC) as the passive method is milder and preserves the integrity of small, fragile tissue samples (Mortazavi et al., 2019). A key modification we introduced to the 2019 Mortazavi et al. protocol is the inclusion of a centrifugation step after the 10-day incubation phase for all samples. This centrifugation step significantly enhances the infiltration of the clearing solution into the tissue and leads to the removal of residual detergent-rich solution that can prevent uniform transparency leading to clearing related artifacts. After centrifugation, COs and FAs were returned to 37 °C clearing solution for 5 days while MB-COs were returned for 14 days. In both cases, the solution was again changed with pre-warmed solution every 3 days. On the final day of heat-assisted clearing (Day 15 for COs and FAs and Day 24 for MB-COs) the samples were centrifuged again and then transferred to pre-warmed TBS, brought to room temperature and left on the shaker until staining.

Using this protocol, we achieved optical transparency in COs and FAs in under 15 days, and in MB-COs in under 24 days (Figures 1D,E). Following clearing, CO and FA samples were incubated in primary and secondary antibody solutions for one week each. Like the clearing phase, we utilized centrifugation at the start of the primary incubation to facilitate antibody penetration. Figure 1F details the immunohistochemistry timeline for COs and FAs. The organoids were equilibrated in 80% glycerol for refractive index matching before confocal imaging.

Following clearing and staining, we use 2 PELCO glass bottom dishes and Blu Tack^®^ (Figure 1C), a reusable adhesive putty commonly used for temporary mounting and stabilization. To create the imaging chamber, a ring of Blu Tack^®^ is molded onto one glass-bottom dish to form a shallow well. The cleared tissue is placed within this well and immersed in 80% glycerol. A second PELCO glass-bottom dish is then placed on top, creating a stable “sandwich” mount that minimized sample movement during image acquisition while maintaining tissue orientation and immersion in the refractive index matching solution. A circular bubble level is used to make sure the tissue sandwich is leveled prior to imaging. This also allows for both sides and orientation of the glass dishes to be imaged (Figure 1C). Furthermore, this process allowed us to maintain sample stability and image the entire organoid by forgoing the need for classical histological sectioning which inevitably severs neuronal processes. Capitalizing on this preservation of tissue integrity, we successfully visualized a whole cleared day-120 CO stained for Neurofilament-heavy chain axonal marker (NF-H) and the astrocytic marker GFAP, revealing the directionality and spatial distribution of neuronal axons and astrocytes within the intact 3D architecture (Figure 1G).

### 3.2 Visualization of cellular heterogeneity in COs using CLARI-O

One of the caveats of classical CO models is a limited heterogeneity and the lack of specific glia cells, such as oligodendrocytes and microglia. Recent advancements allow us to overcome these limitations (Madhavan et al., 2018; Marton et al., 2019). By introducing PDGF-AA, IGF, and T3, we successfully expanded the glial progenitor pool and directed their differentiation into oligodendrocyte progenitor cells (OPCs) and mature oligodendrocytes within dorsally-derived oligodendrocyte-containing COs (dOCOs) (Madhavan et al., 2018). We applied CLARI-O to clear day 120 dOCOs and immunostained them for neuronal nuclei (NeuN), neuronal soma marker, microtubule-associated protein 2 (MAP2), labeling dendrites, and myelin basic protein (MBP), a marker of mature oligodendrocytes. Confocal imaging and 3D reconstruction revealed that oligodendrocytes were distributed in discrete, patchy clusters throughout the dOCO (Figures 2A,B). This distribution likely reflects the clonal expansion of distinct glial progenitor niches that cannot be clearly observed in MBP-stained dOCO sections.

**Figure 2:**
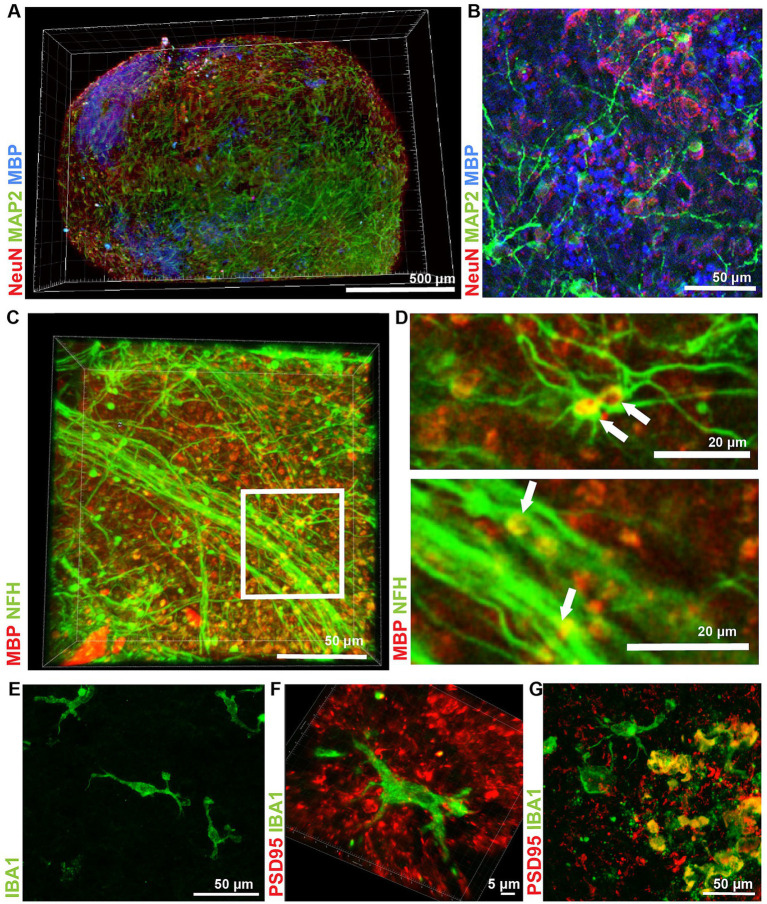
CLARI-O three-dimensional imaging of human COs reveals neuronal, myeline basic protein positive oligodendroglial, astrocytic, and microglial organization. **(A)** Volumetric reconstruction of a CLARITY-O day 150 OCO immunolabeled for MAP2 (green; dendritic, neuronal marker), MBP (blue; myelin basic protein), and neurons (red). The 3D rendering demonstrates extensive neuronal architecture with interspersed MBP-positive oligodendroglial niches throughout the organoid volume. Scale bar, 500 μm. **(B)** Higher-magnification view with inset highlighting a localized pool of MBP-positive oligodendrocytes within the parenchyma. Scale bar, 50 μm. **(C)** Low-magnification view of co-labeling for NFH (axonal marker) and MBP demonstrates MBP-positive oligodendrocytes in close association with NFH-positive axonal fibers (white arrows), consistent with axon–glial interactions within the CO. Scale bar, 50 μm. The boxed region indicates the area shown at higher magnification in the insets in **D**. **(D)** Upper inset: Higher-magnification view demonstrating an MBP-positive oligodendrocytes (arrows) that associate with nearby NFH-positive axons. Individual axons can be resolved as they become ensheathed by oligodendrocyte processes, consistent with active myelination. Lower inset: High-magnification view of a parallel axonal bundle composed of NFH-positive axons (green), with MBP-positive oligodendrocytes positioned within the tract (arrows). **(E)** IBA1-positive microglia (green) incorporated into day 120 CO 7 days prior to fixation. Scale bar, 50 μm. **(F,G)** IBA1-positive microglia (green) and PSD95 (red) labeling demonstrate microglial proximity to synaptic structures, suggesting active microglial–synaptic interactions with synaptic material and a functional immune surveillance state. Scale bar, 5 μm in **F** and 50 μm in **F**.

Furthermore, to investigate axon-glia interactions, we co-stained for NF-H. 3D reconstruction demonstrated colocalization between MBP + oligodendrocytes and NF-H + axons, indicating points of oligo-axonal contact and potential ensheathment or myelination (Figure 2C, white arrows). Higher-magnification views (Figure 2D) reveal oligodendrocyte processes directly ensheathing individual axons, as well as MBP-positive cells positioned within organized axonal bundles, consistent with active, coordinated myelination.

Conventional 2D histological approaches are limited in their ability to capture the spatial extent required to follow long axonal processes and the full scope of oligodendrocyte interactions along them. In contrast, CLARI-O preserves the intact 3D architecture, enabling visualization of axon–glia relationships across extended volumes. Notably, even though we detected MBP-positive NF-H colocalized oligodendrocytes, this spatial overlap was relatively modest (Figures 2C,D). Given that microglia originate from the mesoderm, which is distinct from the neuroectodermal lineage of cortical organoids, they are not typically found in COs (Zhang et al., 2023). Recent studies showed that the introduction of microglia into organoids shaped the neuronal activity of the organoids, and emphasized the critical role of microglia in shaping neuronal activity through synaptic surveillance and refinement (Fagerlund et al., 2021; Butovsky and Weiner, 2018). Considering their crucial role, we separately differentiated iPSC into microglia and subsequently incorporated iPSC-derived microglia into COs (Schafer et al., 2023; Abud et al., 2017). COs were fixed 7 days following the incorporation and processed using CLARI-O. Immunohistochemical staining for microglia (IBA1) followed by confocal 3D reconstruction, demonstrated the effective infiltration of microglia into the CO (Figures 2E–G; Supplementary Movies 1 and 2). Crucially, we observed a significant colocalization between IBA1 + microglial processes and PSD-95 + synaptic puncta, indicating that these microglia actively interact with the synaptic material (Figures 2F,G). Importantly, conventional sectioning often obscures full microglial morphology, frequently severing fine processes depending on the cutting plane. This limitation is especially pronounced in COs, where microglia are sparse, unevenly distributed, and their locations are unknown prior to sectioning, making intact capture challenging. In contrast, CLARI-O as a section-free approach enables visualization of multiple intact microglia with diverse morphologies (Figures 2E–G) and allows for the visualization of their physical interaction with synaptic elements in a 3D environment.

### 3.3 Section-free visualization of forebrain assembloid fusion and interneuron integration using CLARI-O

Beyond single COs, we applied CLARI-O to visualize the complex architecture of forebrain assembloids (FAs) generated by the fusion of ventral (vCO) and dorsal (dCO) organoids. While the migration of GABAergic interneurons and oligodendrocytes across these boundaries is well-documented, the specific structural mechanisms facilitating this fusion and migration remains poorly understood.

Immunostaining of CLARI-O-processed FAs for NeuN and GFAP revealed, for the first time, the existence of GFAP+ parallel fibers specifically located at the junction between the dCO and vCO (Figures 3A,B). These fibers appear to serve as a scaffold, providing structural support for migrating NeuN+ neurons (white arrows in Figure 3B). The presence of these glial bridges at the interface of two tissues is highly reminiscent of glial scar formation observed following CNS injury (Adams and Gallo, 2018). To our knowledge, this is the first detailed characterization of the structural components—specifically GFAP+ fibers, which may be required for physical assembloid fusion.

**Figure 3:**
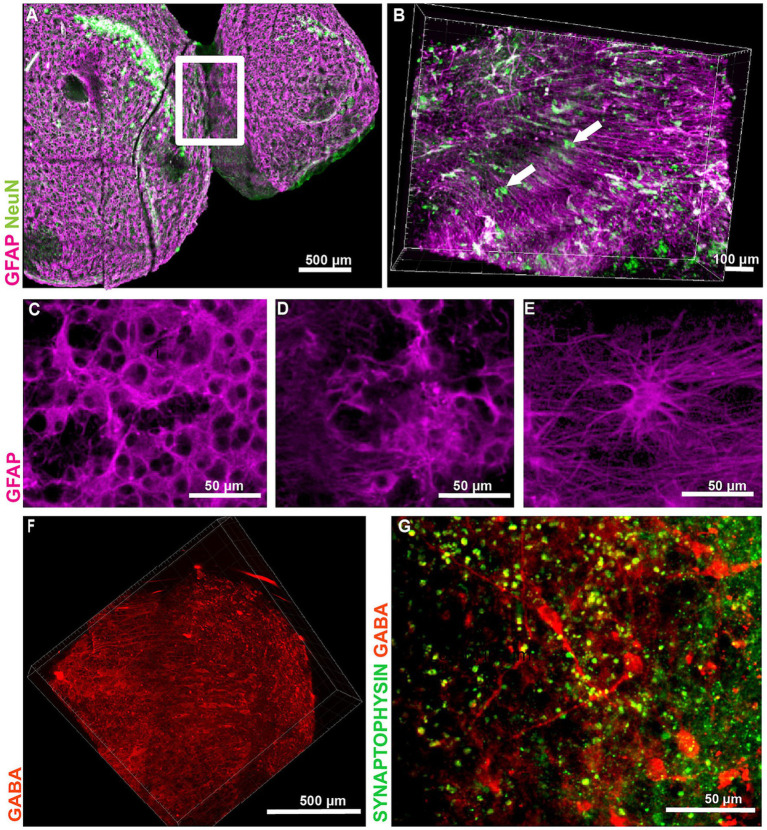
Three-dimensional CLARI-O imaging of human forebrain assembloids (FAs) reveals neuronal migration, astrocytic heterogeneity, and GABAergic network integration. **(A)** Low-magnification view of a CLARI-O-cleared day 120 FA immunolabeled for NeuN (green; mature neurons) and GFAP (magenta; astrocytes). The composite image demonstrates large-scale neuronal distribution embedded within an extensive astroglial scaffold. Scale bar, 500 μm. **(B)** Higher-magnification rendering of the boxed region in **(A)**, showing NeuN-positive neurons (white arrows) interspersed within and migrating along GFAP-positive astrocytic fibers, consistent with astroglia-guided neuronal migration within the FAs. Scale bar, 100 μm. **(C–E)** Representative images illustrating three distinct astrocytic morphologies observed within the assembloid: **(C)** densely packed, stellate astrocytes with overlapping processes; **(D)** transitional astrocytes with elongated somata and moderate branching; and **(E)** highly ramified astrocytes with long, radially oriented processes forming a fibrous network. Scale bars, 50 μm. **(F)** Three-dimensional rendering of GABA-positive interneurons and their neurites within the assembloid, demonstrating extensive process outgrowth and migration across the tissue volume. Scale bar, 50 μm. **(G)** High-magnification image of GABA-positive cells (red) exhibiting developed neuronal morphology and complex arborization. Synaptophysin (green) labels presynaptic puncta; regions of overlap suggest synaptic vesicle compartments potentially containing GABA, consistent with functional maturation of inhibitory circuits. Scale bar, 50 μm.

In addition, we also visualized a morphologically distinct population of astrocytes within FAs. As shown in Figures 3C–E, astrocytes exhibit marked heterogeneity in soma size, process length, and branching architecture, reflecting the emergence of territorial organization and ongoing astrocytic maturation (Ge and Jia, 2016). We identified dense mesh-like networks of closely packed astrocytes with thick, highly branched processes and substantial overlap, consistent with immature or early protoplasmic-like states (Figure 3C); transitional astrocytes with enlarged somata, thicker processes, and partial radial organization suggestive of intermediate maturation (Figure 3D); and stellate astrocytes characterized by well-defined somata and long, thin, radially oriented processes extending across broader domains, indicative of advanced structural complexity (Figure 3E) (Torres-Ceja and Olsen, 2022; Lattke and Guillemot, 2022).

FAs serve as an ideal model for studying inhibitory GABAergic interneurons, the majority of which originate in the ventral telencephalon and migrate dorsally to populate the cortex, hippocampus, and amygdala (van Velthoven et al., 2025; Fazzari et al., 2020). These interneurons are critical for cortical development, as they refine neuronal circuitry and modulate spontaneous synchronous activity (Opitz et al., 2002; Voigt et al., 1997). Using CLARI-O together with immunohistochemistry, we visualized GABAergic interneurons integrated within the surrounding synaptic circuitry, where synaptophysin (SYN^+^) vesicles were frequently associated with GABA^+^ neurites and somata, suggesting functional incorporation of inhibitory neurons into the developing network (Figures 3H,G).

### 3.4 Whole-brain clearing enables 3D visualization of xenograft maturation and integration within the mouse cortex

Absence of vasculature and limited oxygen and nutrient delivery as well as restricted neuronal maturation are some of the limitations of organoid models *in vitro*. To address these challenges, we and others have developed and optimized xenotransplantation of COs into the rodent brain (Wilson et al., 2022; Revah et al., 2022). When engrafted into the mouse retrosplenial cortex (Figure 4A, Ai), COs become vascularized by host vasculature, achieve structural and functional integration, and display robust morphological and functional maturation (Wilson et al., 2022). Clearing xenotransplanted COs within the intact mouse brain is therefore critical for obtaining a comprehensive view of their spatial organization, holistic visualization of cell migration away from the implantation site, and vascular integration.

**Figure 4:**
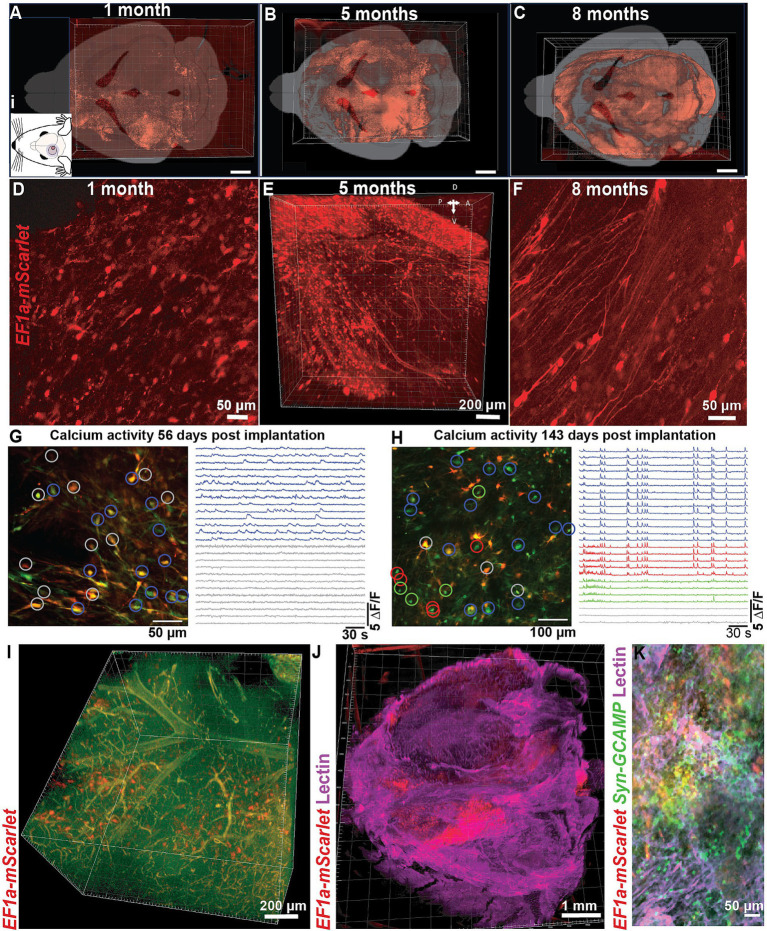
Long-term survival, integration, functional maturation, and vascularization of MB-COs visualized with CLARI-O. The composite image demonstrates **(A–C)** volumetric three-dimensional reconstructions of MB-COs virally labeled with *EF1a-mScarlet* (red) showing the spatial distribution of cells within the host brain at 1 month **(A)**, 5 months **(B)**, and 8 months **(C)** after implantation. Renderings from different post-transplantation time points illustrate progressive growth, structural integration, and spatial distribution of graft-derived cells within the host tissue. Scale bars, 1 mm. **(D)** Higher-magnification view of the graft region at an early post-transplantation stage showing mScarlet-positive human cells concentrated near the graft core with relatively simple cellular morphologies characterized by short neurites and limited branching. Scale bar, 50 μm. **(E,F)** Higher-magnification views at later post-transplantation stages showing increased dispersion of graft-derived cells throughout the surrounding host tissue. Cells exhibit pronounced morphological maturation with elongated neurites, increased branching complexity, and more elaborate neuronal arborization. Scale bar, 200 μm **(E)** and 50 μm. **(G,H)** *In vivo* calcium imaging of spontaneous neuronal activity using GCAMP8s-labeles human CO-derived neurons 56 **(G)** and 143 **(H)** days after transplantation. Left panels show field of view with individual neurons outlined; representative ΔF/F calcium traces are shown on the right, Scale bar, 50 μm **(G)** and 100 μm **(H)**. Calcium imaging at 143 days post implantation demonstrating increased neuronal activity and more synchronized network dynamics among transplanted ne5urons **(H)** as compared to the earlier time point **(G)**. **(I)** Three-dimensional reconstruction of the graft region demonstrating extensive host vascularization surrounding and penetrating the transplanted tissue, visualized through intrinsic vascular autofluorescence. Scale bar, 200 μm. **(J)** Whole-graft volumetric reconstruction demonstrating vascularization of the transplanted CO using lectin labeling (magenta) of host vasculature. Human graft cells are labeled with EFa1a-mScarlet. Scale bar, 1 mm. **(K)** High-magnification image showing transplanted human CO-derived neurons labeled with *Syn-GCaMP* (green) and *mScarlet* (red) and vasculature labeled with lectin (magenta). Scale bar, 50 μm.

Here, we demonstrate that intact whole mouse brains can be safely and reproducibly processed using CLARI-O, following the workflow outlined in Figure 1A. One of the key advantages of CLARI-O is its compatibility with endogenous fluorescent reporters and live-cell imaging workflows. Xenotransplanted COs labeled prior to transplantation with mScarlet under a ubiquitous promoter via lentiviral transduction (LV-EF1alpha-mScarlet) retained robust fluorescent signals at 1, 5, and 8 months post-xenotransplantation (Figures 4A–F). This signal remained readily detectable by confocal microscopy following CLARI-O processing.

Importantly, CLARI-O enables whole-brain visualization of how xenotransplanted CO cells structurally integrate and disperse within the host brain over time. By combining CO xenotransplantation with whole-brain CLARI-O clearing and volumetric imaging, we were able to directly track the spatial distribution and morphology of human CO-derived cells throughout the intact mouse brain. At 1-month post-transplantation, mScarlet-positive human cells were largely confined to the implantation site and exhibited relatively immature morphologies, characterized by short neurites and limited branching (Figures 4A,D; Supplementary Movies 3, 4). In contrast, by 5 months post-transplantation, CO-derived cells were markedly more dispersed, migrating well beyond the graft core and in some cases reaching distant regions, including the contralateral hemisphere (Figures 4B,E,F) and cerebellum at 8 months (Figure 4C). These later stages were accompanied by pronounced morphological maturation, with transplanted cells displaying elongated neurites, increased branching complexity, and elaborate arborization patterns consistent with advanced neuronal differentiation and integration into host circuitry (Figures 4E,F). Together, these data demonstrate that whole-brain CLARI-O imaging provides a powerful platform to map the large-scale migration and maturation of transplanted human neurons within the intact host brain.

To examine the relationship between structural maturation and functional integration following xenotransplantation, we performed longitudinal assessments of neuronal activity using calcium imaging with the GCaMP8s sensor *in-vivo* prior to perfusion. Like the mScarlet labeling strategy, COs were transduced with lentivirus to drive neuron-specific expression of GCaMP8s under the synapsin promoter (LV-hSyn1-GCaMP8s). Consistent with our prior work, this approach enables longitudinal monitoring of calcium dynamics in transplanted neurons for up to 8 months post-transplantation, using a range of *in vivo* imaging modalities (Wilson et al., 2022; Herrema et al., 2025).

Using two-photon (2P) microscopy, we quantified fluorescence changes in neuronal somata and extracted single-cell activity traces, which were expressed as ΔF/F (Herrema et al., 2025). As shown in Figure 4G, at 56 days (~1 month) post-transplantation, GCaMP8s-labeled neurons exhibited largely asynchronous and disorganized calcium transients. In contrast, as seen in Figure 4H, by 143 days (~ 5 months) post-transplantation, neuronal activity displayed a marked increase in synchrony across most recorded neurons, indicative of progressive functional maturation and network-level integration (Trujillo et al., 2019). These functional measurements complement spatial and structural changes observed through CLARI-O in human transplanted CO-derived neurons detected with mouse cortex.

Finally, we used CLARI-O processed MB-COs to visualize the extent of graft vascularization within the mouse cortex. Vascularization was assessed using an autofluorescence-based approach previously validated in zebrafish (Mortazavi et al., 2019; Zeng et al., 2014) (Figure 4I), as well as by lectin staining performed on MB-COs at 8 months post-transplantation (Figures 4J,K). Lectin binds to sugar residues present on endothelial cells and serves as a marker of vascular structures (Battistella et al., 2021). Confocal imaging revealed extensive vascularization of the graft by the host mouse vasculature. Figure 4K shows human, mScarlet and GCAMP-labeled cells embedded within the mouse vasculature (stained with lectin). Collectively, we demonstrate the strength of CLARI-O as a platform for resolving graft vascularization and human cell integration within the mouse cortex in an intact three-dimensional context.

## 4 Discussion

Traditional 2D cell cultures lack the spatial and structural complexities present in living tissues, making them insufficient for studying realistic cellular interactions. In 3D models, cells preserve the spatial cues and cell-matrix interactions necessary to maintain their natural structures, polarities, and behaviors. Human iPSC-derived COs have been invaluable in modeling brain structures and cellular interactions in healthy and disease-associated brain. However, despite these advances, structural and phenotypic analyses following functional assessments, still rely on slicing fixed, frozen COs for subsequent immunostaining. This approach undermines the benefits of the 3D architecture by reducing the data into 2D slices, disrupting spatial orientation, and severing axonal and dendritic projections.

To address these limitations, in the present study, we introduce CLARI-O, an optimized clearing workflow specifically adapted for COs, forebrain assembloids (FAs), and mouse brains containing xenotransplanted organoids (MB-COs). CLARI-O incorporates low-speed centrifugation and sustained heat-assisted passive clearing, a combination that, to our knowledge, has not been systematically applied to human brain organoids or assembloids. This approach meaningfully accelerates lipid extraction while minimizing mechanical disruption, enabling highly consistent clearing across samples of varying size and developmental complexity. The brief centrifugation step helps remove residual detergent-rich solution trapped within the samples, thereby improving the uniformity of transparency and reducing clearing artifacts that commonly arise in spherical organoids. Maintaining all clearing solutions at 37 °C prevents fluctuations in lipid solubilization kinetics (Mortazavi et al., 2019), which is particularly important for organoids, whose dense cytoarchitecture can hinder passive diffusion. This centrifugation-enhanced, heat-stabilized passive clearing protocol standardizes and streamlines the clearing process while preserving delicate 3D architecture.

Building on this technical advance, using post-CLARITY immunostaining and high-resolution confocal imaging, we provide a step-by-step approach to visualize specific cellular structures as well as neuronal and glial cell types. Furthermore, we extended our protocol to MB-COs to enable the visualization of the integration of human cells into the host brain at different stages and extensive vascularization of the xenotransplanted COs within the mouse brain.

To further evaluate the versatility of CLARI-O, we applied the protocol to COs containing additional glial populations, such as oligodendrocytes and microglia. We generated dOCOs containing oligodendrocytes using our previously published differentiation protocol (Madhavan et al., 2018; Li et al., 2022) that targets a small endogenous population of oligodendrocyte progenitor cells and expands their pool over time. Visualization of CLARI-O-processed and immunolabeled dOCOs revealed a patchy distribution of MBP-positive oligodendrocytes, consistent with their derivation from a limited number of glial progenitors. Although MBP-positive oligodendrocytes were readily detectable in day 150 dOCOs, their colocalization with axonal markers is relatively limited, suggesting a potentially constrained myelination capacity within this type of model and a more prolonged organoid maturation that may be necessary to achieve more robust and extensive myelination. Imaging of incorporated microglia enabled high-resolution visualization of their morphology and revealed microglia–synapse interactions, supporting their role in synaptic refinement.

Beyond characterizing individual cell populations, CLARI-O also enabled the visualization of cellular interactions within more complex organoid models, such as assembloids. Assembloid systems have emerged as powerful tools for modeling interregional connectivity, cellular migration, and circuit assembly in the developing brain (Birey et al., 2022; Kim et al., 2025; Izhiman et al., 2026; Wu and Nowakowski, 2025; Kanton and Pasca, 2022; Chen et al., 2020). Previous studies showed multimodal migration of ventral organoid–derived interneurons across fusion interfaces (Bagley et al., 2017) and it has been proposed that the alignment of the progenitor zones at the interface of the fused organoids provides a permissive migratory environment (Bagley et al., 2017; Birey et al., 2017). Using CLARI-O-processed FAs, we visualized, for the first time, continuous radial glia GFAP-positive scaffolds spanning the fusion interface. In conjunction with previous works that show axon-level integration and migration of oligodendrocytes and interneurons through the fusion midline (Birey et al., 2017; Xiang et al., 2017; Kim et al., 2019), our findings provide structural evidence for a glial substrate that may facilitate inter-organoid cellular migration and integration. We also demonstrated a morphologically heterogenous population of astrocytes spanning different developmental stages.

A major advance of this study is the successful clearing of entire mouse brains containing fluorescently labeled transplanted human organoids. We visualized the integration of the human cells into mouse tissue at 1, 5, and 8 months post-surgery. Following the established pipeline to monitor neuronal activity over time using a GCAMP8s calcium sensor, we showed the increased synchrony of neuronal firing over time. In intact human brain, this synchrony picks up before birth and is critical for the proper development of neuronal networks and enhances activity-dependent synaptic refinement (Corlew et al., 2004; Spitzer, 2006; Pires et al., 2021). This shift in spontaneous neuronal activity was accompanied by increased morphological complexity of human neurons, as well as their dispersion and migration within mouse cortex. Moreover, CLARI-O-based imaging revealed a substantial expansion of fluorescently labeled human neurons over time, with some cells potentially acquiring callosal identities and projecting to the contralateral hemisphere. These structural findings should be complemented in future studies by immunohistochemical staining to refine cell-type identification. Finally, lectin labeling further demonstrated robust vascularization of the xenotransplanted COs, consistent with prior observations of extensive vascular integration *in vivo* (Wilson et al., 2022; Revah et al., 2022; Herrema et al., 2025). The dense vascular network of host-derived blood vessels is likely essential for long-term graft survival, integration, and maturation, since it supports the supply of oxygen and nutrients, promotes metabolic exchange, and facilitates the maturation of CO-derived neurons. Our results not only validate successful host-graft integration but also emphasize the need for imaging approaches of capturing intact 3D tissue architecture.

To our knowledge, only few studies applied clearing protocol to assess phenotypic and structural properties of brain organoids, and none has presented a protocol suitable for clearing of MB-COs. Two studies utilized CLARITY, an acrylamide-based hydrogel embedding approach in combination with light-sheet microscopy (Shnaider and Pristyazhnyuk, 2021; Sakaguchi et al., 2019). Sakaguchi et al. (2019) focused on calcium transients in cerebral organoids and used light sheet miscopy and CLARITY clearing to show the presence of ventricular zone (VZ)-like structures. However, the study provided limited methodological detail regarding the clearing procedure itself. Shnaider and Pristyazhnyuk (2021), on the other hand, described a more detailed protocol for CLARITY-based processing, but focused on a single stain of deep layer neurons that required agarose embedding optimized for light sheet microscopy. Most recently, Seo et al. (2017) used microglia-integrated brain organoids to model myelin repair and applied ScaleS4 (Hama et al., 2015), a hydrophilic clearing method that results in a comparatively limited lipid removal. A major drawback of using ScaleS4 is reduced tissue transparency relative to methods that achieve more extensive lipid removal (Hama et al., 2015). Together, these works demonstrate the suitability of tissue clearing approaches for organoid studies, while highlighting, at the same time, the need for more standardized approaches across different experimental systems, including FAs and xenotransplantation models.

Overall, the variety of tissue-clearing methods have been developed, each with distinct strengths and limitations. Hydrogel-based approaches such as CLARITY (Chung et al., 2013) and PACT (Mortazavi et al., 2019; Liu et al., 2017) achieve extensive lipid removal and excellent optical transparency but require additional hydrogel embedding steps and, in some implementations, electrophoretic clearing systems. Solvent-based methods, including 3DISCO (Erturk et al., 2012) and related protocols, can rapidly achieve high levels of transparency but may induce tissue shrinkage, quench endogenous fluorescence, and reduce compatibility with long-term immunolabeling. Hydrophilic methods such as CUBIC (Susaki et al., 2014; Tainaka et al., 2014) and ScaleS4 (Hama et al., 2015) generally preserve tissue morphology and fluorescence well but may require prolonged incubation periods and can achieve less complete delipidation in dense tissues.

CLARI-O incorporates features of multiple clearing strategies to achieve a balance between transparency, tissue preservation, and experimental accessibility. By combining an acrylamide-free SDS-based passive clearing strategy with heat-assisted clearing and centrifugation-enhanced reagent penetration, CLARI-O achieves robust transparency while minimizing tissue handling, specialized equipment requirements, and potential structural distortions. Importantly, the method preserves endogenous fluorescent reporters and supports deep antibody penetration. These features make the workflow particularly well suited for intact COs, FAs, and whole MB-COs. However, CLARI-O is not without limitations. Processing times remain longer than some solvent-based methods, imaging requires substantial computational resources, and optimal imaging depth remains dependent on antibody penetration and microscope configuration. Thus, rather than replacing existing clearing technologies, CLARI-O complements them by providing a practical and accessible workflow specifically optimized for organoid-based systems and xenotransplantation studies. Table 2 provides a summary comparison between these different approaches.

**Table 2:** Comparative strengths and limitations of CLARI-O and existing tissue-clearing approaches.

Method	Lipid removal	Fluorescence preservation	Antibody penetration	Specialized equipment	Organoid compatibility	Xenograft compatibility	References
CLARITY	High	Good	Good	Yes	Good	Moderate	Chung et al. (2013)
PACT	High	Good	Good	No	Good	Moderate	Mortazavi et al. (2019) and Liu et al. (2017)
CUBIC	Moderate–High	Excellent	Good	No	Good	Moderate	Susaki et al. (2014) and Tainaka et al. (2014)
ScaleS4	Moderate	Excellent	Moderate	No	Good	Limited	Hama et al. (2015)
3DISCO	High	Poor–Moderate	Limited	No	Moderate	Moderate	Erturk et al. (2012)
CLARI-O	High	Excellent	Excellent	No	Excellent	Excellent	

Beyond technical advantages, an important strength of CLARI-O is its broad applicability across experimental systems. Our described workflow can be straightforwardly transferred to other assembloid models such as corticothalamic assembloids, corticostriatal assembloids, and cortical-spinal-muscle assembloids (Onesto et al., 2024). We also demonstrated for the first time CALRI-O based clearing and staining of xenotransplanted organoids following functional in-vivo analysis using multiphoton imaging. In the future, we will explore the use of CLARI-O to correlate the identity of cells with certain functional behaviors, i.e., calcium activity patterns, with post-mortem structural analyses such as in-situ immunostaining using endogenous fluorescence or vascular structures as landmarks to co-register *in vivo* and post-mortem imaging datasets. This protocol is also compatible with downstream quantitative analyses and can complement multielectrode array recordings, optogenetic stimulation, and other functional assays.

In summary, our CLARI-O provides a robust, scalable, versatile, and accessible platform for high-resolution three-dimensional visualization of organoids, assembloids, and xenografts, bridging the gap between structure and activity and providing an invaluable resource for exploring disease mechanisms, testing drug effects, and developing new therapeutic strategies in next-generation 3D neural models.

### 4.1 Limitations of the study

In the current study we did not perform detailed structural or morphometric quantification of the cleared COs and FAs. This limitation is partly related to the relatively small sample size in this study, particularly for MB-CO samples, which makes quantitative comparisons across time points derived from individual samples or animals challenging. Nevertheless, these clearing and imaging techniques *can* support such analyses, offering a more efficient and accurate approach to 3D quantification that avoids the limitations of traditional 2D histological methods. This method can also be correlated with functional assessments of human neurons to provide insight into their progressive maturation within the neurodevelopmentally supportive environment of the mouse brain (Wilson et al., 2022; Herrema et al., 2025).

In addition, our current implementation is relatively low throughput, as we performed a single round of immunostaining per organoid, whether derived *in vitro* or recovered following xenotransplantation. However, this reflects an experimental choice rather than a technical limitation of the CLARI-O workflow, and iterative staining and re-staining (Woelfle et al., 2023) should be feasible with this approach. The primary constraint in our study was the limited number of fluorescent channels available on our imaging system (three), which restricted multiplexed marker detection. Access to microscopy platforms with expanded spectral capacity would substantially enhance the depth and multiplexing potential of CLARI-O–based analyses.

A further limitation of this study is that the CLARI-O clearing efficiency was evaluated primarily qualitatively rather than by a direct quantitative analysis. Future studies could incorporate measurements such as optical transmittance, imaging depth, antibody penetration depth, and the preservation of endogenous fluorescent labeling. These additional measures can enable objective and consistent comparisons across experimental conditions.

A further practical limitation of volumetric CLARI-O imaging is the substantial imaging and computational infrastructure required to acquire, process, and visualize large-scale 3D datasets from cleared tissues. Because intact COs and MB-COs are imaged without sectioning, optical access through large tissue volumes requires the use of long-working-distance objectives that are compatible with refractive-index–matched clearing media. In addition, whole-sample imaging generates very large datasets due to tiled acquisition across the cleared specimen. For example, a single organoid imaged at 20X magnification typically required ~60 tiled scans (1,024 × 1,024 pixels each), with acquisition times approaching 10 h and datasets of approximately 60 GB. Imaging of MB-COs produced even larger datasets due to the increased tissue volume and higher magnification required to resolve cellular structures. Whole-brain MB-CO scans at 10 × magnification required 324 tiled images (512 × 512 pixels, triple-channel acquisition), resulting in approximately 13 h of imaging and datasets exceeding 68 GB per sample. Processing, stitching, and rendering image volumes of this scale required a custom-built high-performance workstation with expanded RAM, high-capacity storage, and dedicated GPU resources to enable efficient 3D visualization. Although these requirements currently limit throughput and accessibility, rapid advances in imaging hardware, data storage, and GPU-based visualization platforms are expected to substantially improve the scalability of CLARI-O–based volumetric analyses.

## Data Availability

The raw data supporting the conclusions of this article will be made available by the authors, without undue reservation.

## Supplementary material

Supplementary Movie 1 3D reconstructions of microglia incorporated into CO.

Supplementary Movie 2 3D reconstructions of microglia incorporated into CO.

Supplementary Movie 3 3D reconstructions of MB-CO, 5 months after transplantation.

Supplementary Movie 4 3D reconstructions of MB-CO, 5 months after transplantation.
